# A Phase II pilot trial to evaluate safety and efficacy of ferroquine against early *Plasmodium falciparum* in an induced blood-stage malaria infection study

**DOI:** 10.1186/s12936-016-1511-3

**Published:** 2016-09-13

**Authors:** James S. McCarthy, Thomas Rückle, Elhadj Djeriou, Cathy Cantalloube, Daniel Ter-Minassian, Mark Baker, Peter O’Rourke, Paul Griffin, Louise Marquart, Rob Hooft van Huijsduijnen, Jörg J. Möhrle

**Affiliations:** 1 QIMR Berghofer Medical Research Institute, Brisbane, Australia; 2 University of Queensland, Brisbane, Australia; 3 Medicines for Malaria Venture, Route de Pré-Bois 20, 1215 Meyrin, Geneva Switzerland; 4 Sanofi Aventis Recherche Développement, Chilly-Mazarin, France; 5 Mater Health Services, Brisbane, Australia; 6 Q-Pharm Pty Ltd, Brisbane, Australia; 7 Novartis Consumer Health SA, 2 route de l’Etraz, Case Postale 1279, 1260 Nyon, Switzerland; 8 Sanofi Aventis Recherche Développement, Vitry Alfortville, France

**Keywords:** Induced blood-stage malaria (IBSM), Drug discovery, Ferroquine, Malaria, *Plasmodium falciparum*, Pharmacokinetic/pharmacodynamic modelling, Translational models, SSR97193, Ferrochloroquine

## Abstract

**Background:**

Ferroquine (SSR97193) is a candidate anti-malarial currently undergoing clinical trials for malaria. To better understand its pharmacokinetic (PK) and pharmacodynamic (PD) parameters the compound was tested in the experimentally induced blood stage malaria infection model in volunteers.

**Methods:**

Male and non-pregnant female aged 18–50 years were screened for this phase II, controlled, single-centre clinical trial. Subjects were inoculated with ~1800 viable *Plasmodium falciparum* 3D7A-infected human erythrocytes, and treated with a single-dose of 800 mg ferroquine. Blood samples were taken at defined time-points to measure PK and PD parameters. The blood concentration of ferroquine and its active metabolite, SSR97213, were measured on dry blood spot samples by ultra-performance liquid chromatography with tandem mass spectrometry (LC-MS/MS). Parasitaemia and emergence of gametocytes were monitored by quantitative PCR. Safety was determined by recording adverse events and monitoring clinical laboratory assessments during the course of the study.

**Results:**

Eight subjects were enrolled into the study, inoculated with infected erythrocytes and treated with 800 mg ferroquine. Ferroquine was rapidly absorbed with maximal exposure after 4–8 and 4–12 h exposure for SSR97213. Non-compartmental PK analysis resulted in estimates for half-lives of 10.9 and 23.8 days for ferroquine and SSR97213, respectively. Parasite clearance as reported by parasite reduction ratio was 162.9 (95 % CI 141–188) corresponding to a parasite clearance half-life of 6.5 h (95 % CI: 6.4–6.7 h). PK/PD modelling resulted in a predicted minimal parasiticidal concentration of 20 ng/mL, and the single dosing tested in this study was predicted to maintain an exposure above this threshold for 454 h (37.8 days). Although ferroquine was overall well tolerated, transient elevated transaminase levels were observed in three subjects. Paracetamol was the only concomitant treatment among the two out of these three subjects that may have played a role in the elevated transaminases levels. No clinically significant ECG abnormalities were observed.

**Conclusions:**

The parameters and PK/PD model derived from this study pave the way to the further rational development of ferroquine as an anti-malarial partner drug. The safety of ferroquine has to be further explored in controlled human trials.

*Trial registration* anzctr.org.au (registration number: ACTRN12613001040752), registered 18/09/2013

**Electronic supplementary material:**

The online version of this article (doi:10.1186/s12936-016-1511-3) contains supplementary material, which is available to authorized users.

## Background

Despite medical progress, malaria still ranks among the deadliest infectious diseases, causing nearly half a million deaths among the estimated 214 million cases in 2015 [1]. Malaria is a World Health Organization (WHO) global development priority, and the WHO has shifted its focus from disease control to elimination. Lessons from the past have taught us that progress towards this goal entails both the judicious stewardship of existing treatments and a steady stream of new drugs [2–5]. *Plasmodium* parasite resistance against earlier anti-malarials, chloroquine, amodiaquine and sulfadoxine/pyrimethamine, is now widespread. Artemisinins, the most potent and rapidly-acting anti-malarial agents available today [6], are associated with high recrudescence rates when used as monotherapy, and must be used in combination with other drugs. Worryingly, artemisinin resistance is now seen in the Greater Mekong Subregion [7–10]. Thus, new drugs with new mechanisms of action are urgently needed. Over recent years a number of new, exciting anti-malarial candidates have emerged [4]. The challenge is to prioritize these for further clinical development and to define their pharmacokinetic (PK) and pharmacodynamic (PD) properties in detail, allowing decisions on optimal dosing and the rational choice of partner drugs in a cost-effective and timely procedure.

The experimentally induced blood stage malaria (IBSM) infection model [11] has facilitated the development of new treatments for malaria. In this model, volunteers are inoculated with *Plasmodium falciparum*-infected red blood cells. The model allows measuring critical PK and PD parameters as well as drug safety information in a controlled clinical setting, with parasitaemia that is orders of magnitude below those associated with severe malaria symptoms. The model has already been used with well-established anti-malarials (piperaquine [12, 13] and mefloquine [14]) and experimental drugs (unpublished observations, McCarthy et al. [12]) allowing for benchmarking and uniform PK/PD modelling, accelerating dose finding and synergistic drug partner selection.

Ferroquine (SSR97193, ferrochloroquine) is a drug in development derived from the 4-aminoquinoline chloroquine. Its structure consists of a chloroquine backbone conjugated with ferrocene [15–18]. In vitro studies have shown that the drug has the potential to overcome parasite resistance against chloroquine and other drugs [19–22]. Ferroquine’s main metabolite is *N*-monodesmethyl ferroquine (*N*-desmethyl-ferroquine, or SSR97213), which has similar in vitro activity as ferroquine on chloroquine-sensitive *P. falciparum* strains [16]. Ferroquine has shown good activity against a number of clinical *Plasmodium* isolates [23–25], and in preclinical *Plasmodium berghei* and *Plasmodium vinckei* mouse malaria models [26, 27]. In a Phase I trial the compound was well tolerated at doses up to 1600 mg (single dose) and up to 800 mg as a repeat dose [28]. Ferroquine was recently tested as monotherapy in patients, and combined with artesunate, in a Phase II trial conducted in four African countries [29, 30]. A 3 days combination treatment with doses of 2, 4 and 6 mg/kg/day ferroquine and 4 mg/kg/day artesunate resulted in 97–99 % cure rates, whereas the 4 mg/kg ferroquine monotherapy produced a 79 % cure rate.

To further develop ferroquine for the treatment of malaria a thorough understanding of the drug’s PK and PD properties is necessary. Therefore, a single-dose, phase II trial was performed in the human malaria model. Healthy volunteers were inoculated with infected erythrocytes and treated with a single dose of ferroquine. The principal aim of the current study was to establish an accurate PK/PD model for ferroquine in the IBSM model, so as to guide further dosing and selecting optimal partner drugs.

## Methods

### Study design and population

This was a single-centre phase II, clinical trial (registration number ACTRN12613001040752). It was conducted between October 17 and December 11, 2013 at Q-Pharm Pty Ltd in Australia. The primary objective was to characterize the PK/PD relationship of ferroquine on clearance of *P. falciparum* from the blood in volunteers following experimental *P*. *falciparum* infection. The secondary objective was to characterize the PK profile of ferroquine in this model, as well as to assess the drug’s safety and tolerability. The study was conducted in accordance with the principles of the Declaration of Helsinki (1964 and subsequent updates), and with the NHMRC National Statement on Ethical Conduct in Human Research (2007). The conduct of the study was in accordance with the Notes for Guidance on Good Clinical Practice (CPMP/ICH/135/95), as adopted by the Australian Therapeutic Goods Administration (2000). Enrolment included male and non-pregnant female volunteers between 18 and 50 years of age, who met the inclusion criteria and none of the exclusion criteria.

## Study conduct

### Treatments

Subjects were inoculated with ~1800 viable *P. falciparum* 3D7-infected human erythrocytes administered intravenously (single dose) as described previously [11]. The day of inoculation was considered as day 0 for every subject. Subjects were monitored on an outpatient basis for adverse events and any unexpected early onset of symptoms, signs or parasitological evidence of malaria. Volunteers were treated with ferroquine the morning after the parasitaemia measured by quantitative PCR (qPCR) first exceeded a threshold of ≥1000 parasites/mL, or earlier if clinical manifestation of malaria appeared. Subjects were then confined for 48 h for ferroquine administration, determination of parasite load and drug levels, and safety monitoring. A single dose of 800 mg ferroquine was administered to volunteers in a fasting state in 100 mg-capsules (Sanofi France SA). If clinically well, subjects were then discharged and monitored on an outpatient basis for safety, ferroquine/SSR97213 blood levels and the continued presence of malaria parasites as measured by qPCR. The effects of ferroquine on parasitaemia were observed for up to 16 days before compulsory rescue treatment: Rescue treatment on day 16 consisted of artemether/lumefantrine (A/L; Riamet^®^, 20 mg artemether/120 mg lumefantrine; Novartis Pharmaceuticals Australia Pty Ltd.) administered as four tablets every 12 h for 60 h. Rescue treatment would be provided earlier if recrudescence or clinical failure were observed. As a safety measure, parasitaemia continued to be monitored following the A/L therapy and was again assessed at the end of study visit, which took place on day 28. If gametocytaemia was detected, as determined by quantitative qPCR for the female gametocyte-specific transcript *pfs25*, subjects were treated post-A/L treatment with a single 45 mg dose of primaquine, administered in tablets each containing 13.2 mg primaquine phosphate (equivalent to 7.5 mg primaquine; Boucher and Muir Pty Ltd).

### Measurement of ferroquine/SSR97213 blood concentration

Concentrations of ferroquine and SSR97213 were measured in whole blood. Blood samples were taken pre-dose and at 0.5, 1, 2, 4, 6, 8, 12, 24, 48, 72, 96 and 144 h post ferroquine dosing; then on days 8, 11, 14 and 28 (End of the Study). The blood was collected in EDTA tubes, then processed and spotted onto dried blood spot (DBS) cards. Ferroquine and SSR97213 plasma concentration was measured in DBS samples by UPLC column separation with reversed-phase chromatography followed by detection with triple-stage quadrupole MS/MS in the selected reaction monitoring mode. This assay was validated in a separate study using [^2^H_6_]-ferroquine and [^2^H_3_^13^C]-SSR97213. In summary, sensitivity for both was 5–1000 ng/mL for 10 mL injections, with 3.5–5.1 and 2.6–6.4 % intra-run precision (n = 20, four concentration levels), with accuracies of 98.6–104 and 97–102 % for ferroquine resp. SSR97213.

### Measurement of parasitaemia

Blood samples were taken to quantify parasitaemia by qPCR as described [31]. Post inoculum, blood samples were taken daily, then twice daily after a positive PCR signal was obtained. Upon ferroquine administration (after reaching the 1000 parasites/mL threshold) samples were taken at 0, 2, 4, 8, 12, 16, 20, 24, 30, 36, 48, 60, 72, 84, 96, 120 and 144 h post ferroquine dosing, then three times a week until two consecutive negative PCR values were observed. Blood samples were also taken on the morning of the first A/L dose and the two subsequent mornings; pre- and post any primaquine dose and then at the end of study visit. Whole-blood samples were stored on ice. The detection limit was 64 parasites/mL. *P*. *falciparum* gametocytaemia was quantified using a previously described RT-PCR assay specific for *pfs25*, a *P. falciparum* mRNA transcript expressed in mature female gametocytes [13].

### Safety assessments

At screening, all subjects underwent a complete physical examination, monitoring of vital signs, ECG and routine biochemistry and haematology testing. During confinement, vital signs were measured three times per day and all symptoms and adverse events were recorded per protocol (see Additional file 1). Blood samples were assayed for a number of haematological abnormalities, including total protein, globulin, aspartate transaminase (AST), alanine transaminase (ALT), lymphocytes, neutrophils, white cell count, platelets, haemoglobin, haematocrit, red blood cell count, haemolysis index, ferritin, iron, LDH, creatine kinase, sodium, potassium, chloride, albumin, urea, total iron binding capacity (TIBC), cholesterol and HDL ratio, triglyceride, bilirubin, bicarbonate and alkaline phosphatase.

### Study outcomes

The outcomes of this study were the PK parameters C_max_, T_max_, t_½_, AUC_t_ and AUC_inf_ of ferroquine and SSR97213; PD parameters, including parasite reduction profile and parasite reduction ratio (PRR); safety monitoring, including 12-lead electrocardiograms (ECG; see Additional file 1).

### Sample size

Historically, a number of eight subjects in a dose cohort has proven sufficient to characterize the effects of a drug on malaria parasite kinetics following induction in healthy volunteers using inoculation with blood stage *P. falciparum* [11, 12]. In this study, one cohort (n = 8) was recruited.

### PK, PD and PK/PD modelling

Non-compartmental PK analysis was performed using WinNonin (Pharsight, Cary, US). The area under the curve was determined using log-linear integration up to last time point measure (AUC_t_) and extrapolated to infinity (AUC_inf_). The gradient of the elimination phase, λ_z_, was determined by log-linear regression leading to calculation of the elimination half-life. The maximal concentration following ferroquine administration (C_max_) and the time it was reached (T_max_) were reported. All parameters were summarized using appropriate statistics including geometric mean or median. Compartmental modelling was performed using NONMEM ([32]; version 6 or higher).

For PD analysis, each subject’s Parasite Reduction Ratio (PRR; 48 h) and parasite clearance half life and corresponding 95 % confidence intervals (CI) were calculated using the slope and corresponding standard error (SE) of the optimal regression model [33]. The weighted average slope estimate and corresponding SE were calculated by the inverse-variance method. Where the model fit was adequate (p < 0.001), subjects’ data were used to estimate the PRR for each dose cohort and the corresponding 95 % CI.

The PK/PD modelling procedures were similar to those in two recent studies [14, 34]. PK/PD analysis was performed and the minimum parasiticidal concentration (MPC) for ferroquine was determined by observational and model-based analysis. MPC is the lowest plasma or blood concentration of a drug that produces the maximal anti-malarial effect [2, 35]. The PK/PD modelling approach was as outlined previously [36, 37], using Nonmem VII (Globomax), R (v2.14) and Berkeley Madonna (University of California) to model and analyse the data. Parameters obtained from the WWARN calculator [38] used default settings with recrudescence values pre-censored.

## Results

### Subjects

Among 19 subjects screened for Cohort 1, eight subjects were enrolled in the study, infected with *P. falciparum* and, upon reaching the predetermined threshold of 1000 parasites/mL (Additional file 1: Figure S1) received treatment with 800 mg ferroquine. Among these subjects, three were males and five females with average age 26 years (±6.4) and body mass index 23.3 kg/m^2^ (±1.7). Seven were of Caucasian origin, one was Asian. All eight subjects completed the study. The initial plan was to recruit two cohorts (n = 8 each) for different doses of ferroquine. However, the second cohort was not recruited, because the key pharmacodynamics parameters were successfully obtained in the first cohort, and the Safety Review Team recommended not to recruit a second cohort after review of safety data (see below).

### Pharmacokinetic analysis

**Fig. 1:**
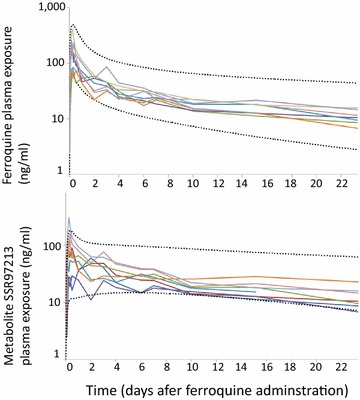
Time course of individual ferroquine and SSR92713 exposure following oral administration of 800 mg ferroquine. Predicted data were presented as the 2.5th and 97.5th confidence intervals using Berkeley Madonna denoted by the *black dashed* time courses. The model (1000 simulations) used an adjusted first order input

The individual plasma exposures to ferroquine and its metabolite over time are shown in Fig. 1. Non-compartmental analysis was performed on the data to determine the PK parameters for the cohort (n = 8), with the results presented in Table 1. The observed censored data supported the fitting of a two-compartment model which could be successfully used in both Nonmem^®^ [32] and Winnonlin^®^ [39, 40] software packages, with a fit that improved when the data were truncated at 264 h. A visual predictive check (Additional file 1: Figure S2) demonstrated that the two-compartment model was sufficient for describing PK data until 240 h. A first-order absorption without a lag phase was the most successful parameterization of absorption.

**Table 1:** Non-compartmental analysis parameter estimates of the exposure of ferroquine and SSR97213

	Ferroquine	SSR97213
C_max_ (ng/mL)		
Geometric mean	155	89
Log-linear CIs	(94–257)	(49–162)
T_max_ (h)		
Median (range)	6 (4–8)	8 (4–12) one subject at 48 h)
C_144 h_ (ng/mL)		
Geometric mean	26	26
Log-linear CIs	(21–32)	(19–36)
Half-life (h)		
Geometric mean	262 (10.9 days)	661 (27.5 days)
Range	(204–315)	(295–1349)
AUC_144 h_ (ng/mL)		
Geometric mean	6893	5525
Log-linear CIs	(5378–8834)	(3883–7861)
AUC_0−last_ (ng/mL)		
Geometric mean	12,406	12,247
Range	(9259–17,826)	(6449–19,975)
AUC_0−Inf_ (ng/mL)		
Geometric mean	16,631	31,006
Range	(11,314–25,091)	(10,061–55,768)

### Pharmacodynamic analysis

**Fig. 2:**
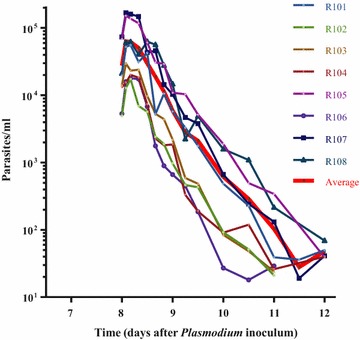
Parasitaemia rise and fall following ferroquine treatment for the individual participants (*red* represents the mean)

The reduction in parasitaemia following ferroquine treatment as reported by the PRR and parasite clearance half-life for the individual participants is shown in Fig. 2 and Table 2. The overall cohort-specific PRR was 162.9 (95 % CI 141–188). Two subjects experienced recrudescence and required A/L treatment at 288 h. For the first of these, the parasitaemia nadir for the testing episode was measured at 132 h (ferroquine dosing was at 72 h); at this time point, the estimated ferroquine exposure was 22.7 ng/mL (the measured values were 31 and 17 ng/mL at 96 resp. 144 h). For the second volunteer, the nadir was estimated to occur between 150 and 160 h, with a ferroquine exposure between 16.4 and 25.2 ng/mL. The cohort parasite clearance half-life was 6.5 (CI 6.4–6.7) h.

**Table 2.** Individual parasite reduction ratios (PRR) and half-life (t½) for 800 mg ferroquine

Subject	PRR (95 % CI)		t½, h (95 % CI)	
R101	73	(47–115)	7.75	(7.01–8.66)
R102	140	(99–198)	6.73	(6.29–7.24)
R103	375	(222–634)	5.61	(5.16–6.16)
R104	244	(129–460)	6.05	(5.43–6.84)
R105	250	(116–542)	6.02	(5.29–7.00)
R106^+^	78	(60–101)	7.64	(7.21–8.13)
R107	124	(69–220)	6.91	(6.17–7.85)
R108	479	(356–646)	5.39	(5.14–5.66)

^+^ Subject R106 had low levels of parasitaemia for a period of time after 144 h post treatment. Data for analysis were censored at 144 h

### Pharmacokinetic/pharmacodynamic assessments

Based on the clearance rate of 0.09 (0.06–0.10) h^−1^ estimated from the WWARN calculator [38], we calculated the time taken for parasitaemia to decrease to an extrapolated intercept with either a threshold of 0.003 or 0.0002 parasites/mL (equivalent to 1 parasite per 70 kg subject) and the equivalent concentration of ferroquine concurrent with these times. The extrapolation indicated that the duration over which the exposure would need to exceed the MPC, on average, had to be greater than 142 h (95 % CI 115–211 h). Integration of the MPC data and testing within trial simulations indicate a MPC of 20 ng/mL (% CV = 20). This analysis predicted that a single 800 mg dose of ferroquine would exceed this MPC for 454 h (37.8 days) in patients, taking into account that clearance is about 32 % lower in patients as compared to healthy subjects, thus leading to higher exposure levels in patients for a given dose.

### Appearance of gametocytes

Results of a PCR-based methodology to investigate for presence of the sexual stage of the parasite that is transmissible to mosquitoes indicated that all eight subjects became positive for the gametocyte-specific transcript *pfs25*. Transcripts were first detected in the blood approximately 7 days after subjects had received ferroquine and remained detectable until subjects received a dose of primaquine at the end of study (See Additional file 1: Figure S3).

### Safety

Of the total of 84 adverse events reported (see Additional file 1: Table S1), 12 were assessed as possibly related to the inoculum (i.e. malaria-related), 42 were assessed as probably related to the inoculum, and seven were assessed as unlikely to be related, or unrelated to inoculation (see Additional file 1: Table S1). Of those adverse events possibly or probably related to malaria, 40 events occurred after the administration of ferroquine and before commencement of A/L, and two within 1 day of the start of A/L. Eight adverse events were assessed as probably, and three events were considered to be possibly related to ferroquine (nausea and transient elevation of transaminase levels). This study was put on hold following the occurrence of these three separate events occurring in three subjects that were classified as serious adverse events (SAEs) by the sponsor. As discussed above, this formed part of the rationale not to undertake a second cohort.

Eight days after inoculation, prior to ferroquine administration, a single subject had clinically significant elevated transaminases of AST 110 U/L and ALT 81 U/L (reference range 10–40 and 5–40 U/L for AST and ALT respectively) that was identified on a *per protocol* biochemical test. Transient derangements of liver function tests (LFTs) of this magnitude are routinely observed in IBSM studies. These changes normalized post confinement for ferroquine treatment, but increased again after end of study treatment with A/L, peaking at levels of AST 208 U/L and ALT 347 U/L on day 28 (day 20 post ferroquine). The relationship of these derangements with the administration of ferroquine could not be determined with certainty, as the ALT peak occurred after the subject had received A/L treatment, which can lead to transient elevation of transaminases [41]. These changes had returned to baseline by day 42. A second subject was found to have significant elevations in transaminase levels (AST 355 U/L and ALT 247 U/L) 3 days after receiving ferroquine (day 11). These peaked on day 12 at 693 and 536 U/L for AST and ALT respectively before returning to near normal ranges by days 21 and 28 and remained normal to day 44. A third subject was also noted to have clinically significant elevation of transaminase levels of AST 252 U/L and ALT 396 U/L 5 days after ferroquine administration (day 13). These parameters returned to near-normal by day 16, but the ALT rose again following A/L treatment to reach a level of 144 U/L on day 19. At the end of study (day 28), the subject’s biochemistry test was within the normal range. Note that volunteers were confined only for 48 h, therefore, non-ferroquine-related factors may have contributed to ALT/AST elevations. As bilirubin levels remained normal at all times, these derangements in liver function were not considered SAEs according to Hy’s Law [42]. Paracetamol was the only concomitant treatment among subjects that may have played a role in the elevated ALT/AST levels, as these were observed in two out of the three subjects in this study who required paracetamol for symptom relief of headaches/fever. In addition abnormal haematology results (neutropenia and/or thrombocytopenia) were reported in these two subjects, preceding the transaminitis event onset, which were attributed to the onset of malaria).

In the absence of the identification of another plausible cause, and with the temporal association following ferroquine administration it was concluded that these adverse events were likely attributable to ferroquine. However, given the concurrence of a range of other potential hepatic irritants (notably malaria, and concurrent medications), it not possible to exclude these as cofactors. No clinically significant ECG abnormalities were observed.

## Discussion

The PRR calculated for ferroquine in this study (163; 95 % CI 141–188) corresponds to the 10–10^3^ range reported earlier for quinine, mefloquine and sulfadoxine-pyrimethamine, and thus is somewhat in the lower range of 10^2^–10^4^ for 4-aminoquinolines and halofantrine, and 10^3^–10^5^ for artemisinins [43]. The present study is expected to be typical for malaria-naïve individuals who would be more susceptible to clinical illness, and further not have clearance estimates elevated by immunity, compared to patients in malaria-endemic areas [44]. PRR variation due to differences between patient and volunteer status was recently highlighted [45]. As reported elsewhere [16], it would therefore seem that the principal benefit of the added ferrocene moiety in ferroquine, compared to its predecessor chloroquine, is its activity against chloroquine-resistant parasites, with a good overall fit to Target Compound Profile 1 as described earlier [2, 46]. In this respect it is important to note that the *P. falciparum* strain tested, 3D7, is chloroquine-sensitive. Possible future use of the chloroquine-resistant challenge strain 7G8 [47] may offer a means of addressing the activity of ferroquine against chloroquine-resistant parasites. The study described here also confirms ferroquine’s attractive in vivo half-life (11 days), which suggests that it may be a suitable partner drug when paired with short half-life and fast-acting drugs including artemisinins and related peroxides, and thereby fit to Target Compound Profile 2 [2]. As the rate of parasite killing is believed to be constant while the drug concentration remains above the MPC, the parasite clearance half-life and PRR can be derived during the initial phase of parasite killing. The parasitaemia levels in volunteers in this experimental infection system are orders of magnitude lower than those seen in patients and it is, therefore, possible that PD effects may be affected by parasite concentration. However, PD data from two recently published studies of mefloquine [14]) and OZ439 [34] in the IBSM model were close to those reported in studies undertaken in patients with clinical malaria [48, 49].

The appearance of gametocytes after anti-malarial treatment is an observation previously made, both in field studies [50] and in IBSM studies [33] and [51]. The fact that the signal persisted in the presence of both ferroquine and its active metabolite suggests that at least mature female gametocytes are not cleared in vivo by ferroquine. Without mosquito transmission data it is not possible to determine if ferroquine-treated subjects would be infectious to mosquitoes. Factors that may influence this include the (unmeasured) activity of the drug against male gametocytes and possible damage to the parasites that may prevent sporogony in the mosquito.

While ferroquine was, overall, well tolerated, three subjects experienced increased transaminase readings. Although transient Grade 1 (WHO) elevations of transaminase are typically observed in IBSM studies, these typically occur at earlier timepoints than was observed in two of the three subjects reported here. In two of the three subjects transaminases peaked 3–5 days after ferroquine administration (at 693 and 536 U/L and 252 and 396 U/L for AST and ALT respectively) before returning to normal values. Intensive investigation, including a measurement of paracetamol levels did not reveal a definitive cause for these changes. Increased transaminase readings had also been seen in four adult patients in the recent ferroquine Phase II trial, which involved 187 patients, again in absence of concomitant abnormal bilirubin readings [29]. That study, too, permitted the use of paracetamol, along with contraceptives, as the only permissible concomitant treatment. Toxic paracetamol (over-)dosing is well known to cause acute liver toxicity but even normal use may result in modestly increased AST and ALT readings in the absence of elevated bilirubin levels [51–54]. Further establishment of ferroquine’s safety profile will require additional clinical trials.

## Conclusions

In conclusion, ferroquine showed adequate anti-parasitic efficacy with clearance of parasitaemia in all eight subjects treated. The parameters derived from the PK/PD model of ferroquine, combined with its long in vivo half-life, indicate that ferroquine has potential in the development of new combination anti-malarial treatments. However, additional clinical trials are needed to evaluate the safety of ferroquine beyond the small number of participants in this study. Subjects should undergo careful liver function monitoring while taking this investigational drug. This study provides important information about the efficacy and tolerability of ferroquine, which would be beneficial for further development of the drug as a partner in a combination treatment.

## Additional file

10.1186/s12936-016-1511-3 McCarthy et al*._*Supplement. Supplementary data for probably related adverse events, detailed transaminase findings, individual parasite growth following inoculation (prior to ferroquine administration), pharmacokinetic model visualization, gametocytaemia and expanded methods for pharmacokinetic/pharmacodynamic modelling.

## Abbreviations

ALT: alanine transaminase
AST: aspartate transaminase
AUC_t_: area under the curve (until time = t)
CI: 95 % confidence interval
DBS: dry blood spot
ECG: electrocardiogram
IBSM: induced blood-stage malaria
LFT: liver function tests
MPC: minimal parasiticidal concentration
PD: pharmacodynamic
PK: pharmacokinetic
PRR: parasite reduction ratio
qPCR: quantitative PCR
SAE: serious adverse event
SE: standard error
TIBC: total iron binding capacity
WHO: World Health Organization
