# Supplementary material for: A Phase II pilot trial to evaluate safety and efficacy of ferroquine against early Plasmodium falciparum in an induced blood-stage malaria infection study
Source: Malar J. 2016 Sep 13;15(1):469. doi: 10.1186/s12936-016-1511-3 (PMC5022189; doi:10.1186/s12936-016-1511-3)
Supplement: Supplementary file 1 — 10.1186/s12936-016-1511-3 McCarthy et al._Supplement. Supplementary data for probably related adverse events, detailed transaminase findings, individual parasite growth following inoculation (prior to ferroquine administration), pharmacokinetic model visualization, gametocytaemia and expanded methods for pharmacokinetic/pharmacodynamic modelling. [file 12936_2016_1511_MOESM1_ESM.pdf]

Supplementary Data

Table S1 : Probably Related Adverse Events

**Probably Related Events Summary**					
Category	**Event**	**Inoculum**	**Ferroquine**	**Riamet**	**Total**
Symptoms	Anorexia *(including Loss of appetite)*	1	0	0	1
	Back Pain	1	0	0	1
	Fatigue *(including Fatigue and Lethargy)*	3	0	0	3
	Gastrointestinal upset *(including Nausea)*	0	4	0	4
	Headache *(including Headache and Intermittent headache)*	13	0	0	13
	Dizziness *(including Lightheaded)*	2	0	0	2
	Myalgia	1	0	0	1
	Rigors	1	0	0	1
	Sweats *(including Intermittent sweating)*	1	0	0	1
Signs	Chills *(including Chills and Intermittent chills)*	3	0	0	3
	Fever	9	0	0	9
Abnormal Laboratory Results	Elevated transaminase *(including Elevated alanine transaminase and Elevated aspartate transaminase)*	2	4	0	6
	Leucopoenia/Neutropenia	2	0	0	2
	Lymphopenia	1	0	0	1
	Thrombocytopenia	2	0	0	2
**TOTAL**		**42**	**8**	**0**	**50**

Transaminase findings

- On Day 8, Subject R101 had clinically significant elevated transaminases of AST 110 U/L and ALT 81 U/L (normal range 7-40 U/L). These parameters settled post confinement for ferroquine treatment but increased again post treatment with Riamet peaking at levels of AST 208 U/L and ALT 347 U/L on Day 28. This transaminitis was considered to be a Serious Adverse Event and was reported accordingly. These parameters returned to baseline by Day 42.

- On Day 11 Subject R104 was also found to have clinically significant elevated transaminases of AST 355 U/L and ALT 247 U/L. These parameters peaked at levels of AST 693 U/L and ALT 536 U/L on Day 12 and returned to within non-clinically significant ranges by Day 21 and Day 28 for AST and ALT respectively. This transaminitis was considered to be a Serious Adverse Event and was reported accordingly. SAE recorded as resolved with liver function tests within normal ranges on Day 44.

- On Day 13 Subject R105 was also noted to have clinically significant elevated transaminases of AST 252 U/L and ALT 396 U/L. These parameters had returned to within non-clinically significant ranges by Day 16 but ALT peaked again on Day 19 at a level of 144 µg/L. This had settled by end of study (Day 28). This transaminitis was considered to be a Serious Adverse Event and was reported accordingly. SAE recorded as resolved with liver function tests within normal ranges on Day 24.

Figures

Fig. S1: Individual parasite growth following inoculation (prior to ferroquine administration).

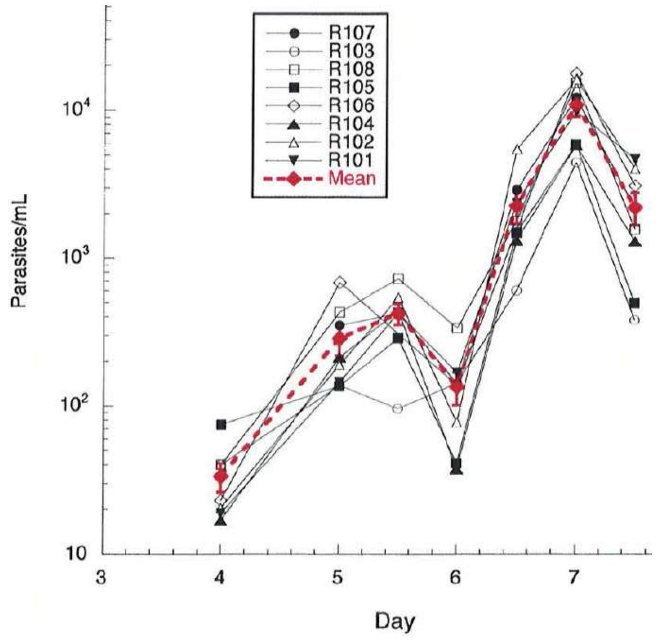

Fig. S2: A visual predictive check of the model's ability to reflect the exposure data. Circles, black lines: observed data; Red lines: Predicted 0.05 and 0.95% prediction quantiles; Grey shading: Predicted 0.25 and 0.75% inter-quantile range. Time is measured from start of ferroquine administration.

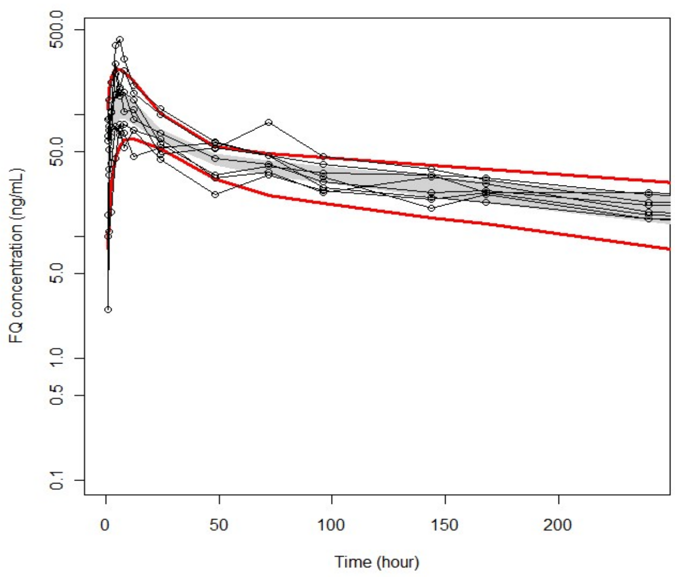

Fig. S3: Gametocytemia, expressed as Pfs25 transcripts, measured as a function of time after ferroquine administration. The time-point at which primaquine was administered is indicated with an arrow.

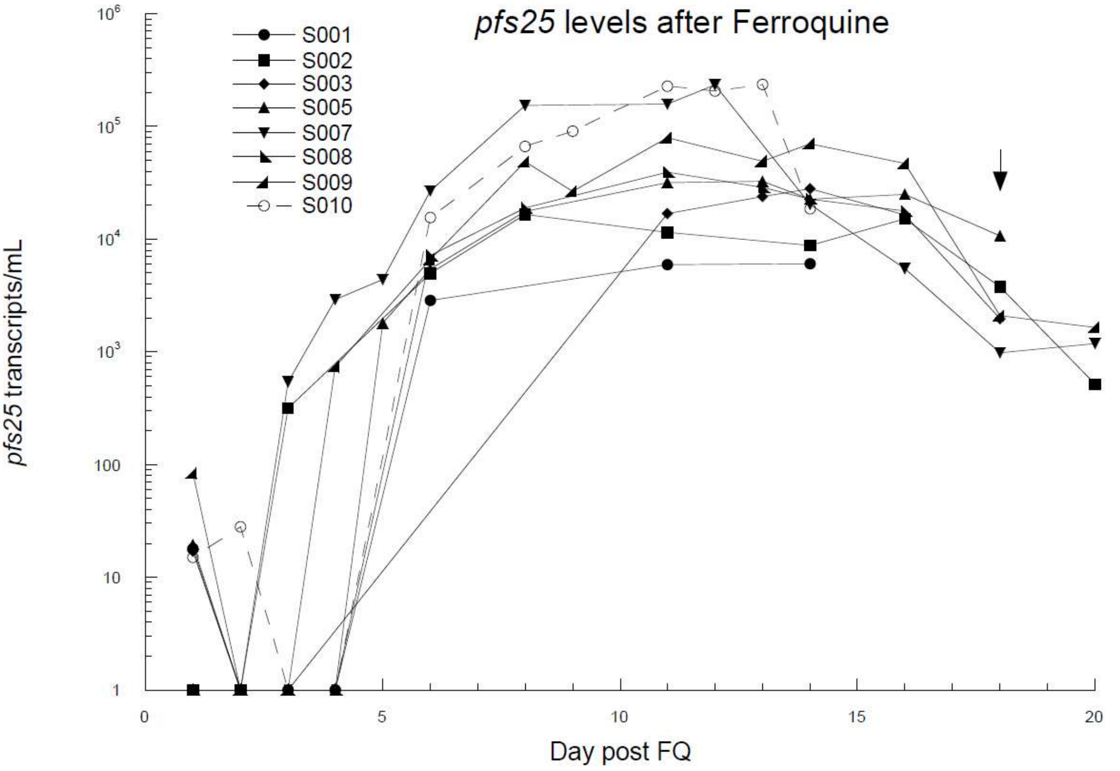

Expanded Methods to PK/PD modelling

Methods

The modelling of pharmacokinetic and parasitemia data was performed in a two-step process:

1. Compiling and fitting a PK model
2. Then using that PK model during the analysis of the PD (parasitemia) data.

Pharmacokinetics

A previously developed three-compartment model was fitted to the ferroquine blood level data. However, both this model as well as additional three compartment models failed to adequately reflect the data (Figure S4).

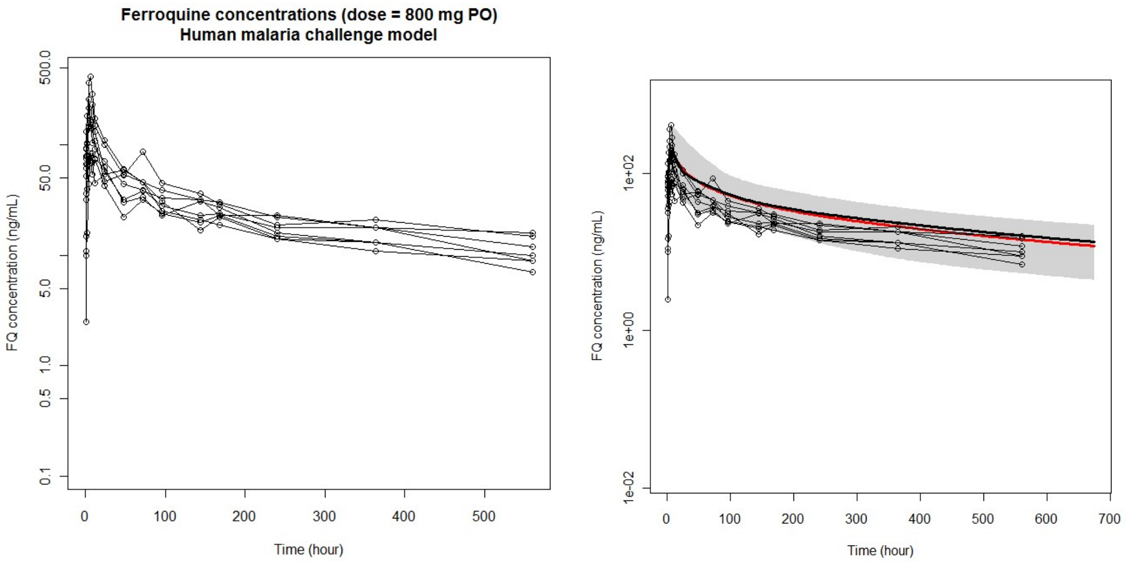

Figure S4 A) Observed ferroquine pharmacokinetic profile following administration of 800 mg PO (full data) and B) simulations of the 3 compartment model (red=median coded in Nonmem, black=median coded in R) overlaid with the observed challenge model data.

The grey shading denotes the predicted range between the 5th and 95th quantiles estimated in R, code = 4.2.
Observed data = points, n=8, Data censored to 240 hours.

Attempts were made to fit a PK model to time-truncated data where the cutoff occurred after the nadir in parasitemia. The parasitemia time courses showed two nadirs, one after ferroquine and one after artemether/lumefantrine (A/L; given for rescue therapy after recrudescence) - Subjects R101 and R106 recrudesced and were treated with A/L at 288 hours following dosing with ferroquine. Subject R101 had an observed nadir of parasitemia at 132 hours after ferroquine dosing (dosing occurred 72 hours after first post-inoculum detection of parasites), coinciding with ferroquine concentrations of 31 ng/mL at 96 hours and 17 ng/mL at 144 hours. The time of parasitemia nadir for subject R106 was estimated as being between 149.5 and 160.0 hours. The remaining volunteers did not display signs of recrudescence or require rescue intervention with A/L. A cutoff of 240 hours was

chosen / implemented because the substantial changes in parasitemia occurred before this point and it was the longest time course where PK modelling was feasible.

The aim of the PK modelling was to accurately capture the pharmacokinetic profile in the volunteers during the period of parasitemia. A pragmatic approach was used where the accurate reflection of the individual PK took precedence over creating a model reflecting the population PK of the molecule – any model would be hindered by the low number of volunteers, the need for truncation and the discrepancy in PK with the pre-existing population PK model for this molecule.

A variety of models were tested including different parameterizations of absorption and application of inter-individual variance (IIV). The absorption models tested included:

- First order
- Zero order
- A mixture of first and zero order
- A lag phase
- Serial absorption compartments [1]

A two compartment model with first order absorption without a lag phase was the most successful parameterization of absorption (Table S2). Other absorption models including zero order, a mixture of first and zero order, a lag phase, and serial absorption compartments [1] were tested but inferior to the first order parameterization.

The model results were:

Parameter	Units	Value (SE)	95% CI
**Obj**		805.9	
**V2/f**	L	1138 (0.43)	(485 - 3686)
**V3/f**	L	10570 (0.14)	(6996 - 15867)
**CL/f**	L/hr	61.4 (0.06)	(46.9 - 70.7)
**Q2/f**	L/hr	284 (0.37)	(180 - 394)
**Ka**	/hr	0.10 (0.43)	(0.06 - 0.26)
**IIV_V2**		0.46 (0.53)	(0.09 - 1.26)
**IIV_CL**		0.06 (0.95)	(0.01 - 0.19)
**IIV_Q**		0.23 (0.79)	(0.05 - 0.62)
**EPS**		0.09 (0.29)	

Table S2 Parameter estimates for the fitting of a two compartment, first order PK model to the ferroquine human challenge model PK data.

N=8, model = run40.mod, method=first order conditional estimation with interaction in Nonmem VII, confidence intervals estimated from bootstrap conducted with 3000 replicates using PsN and Nonmem.

V2/f = central volume of distribution, V3/f = peripheral volume of distribution, CL/f = irreversible clearance from the central compartment, Q2/f= inter-compartmental clearance, Ka= first order absorption rate constant, Obj= objective function, IIV = inter-individual variance, EPS = multiplicative random variance

The *post hoc* Bayesian estimates of the individual volunteers were also reported for use in the PD modelling (Table 2). They adequately reflected the PK in the individual volunteerstable S3.

ID	KA (1/hr)	V2/f (L)	V3/f (L)	CL/f (L/hr)	Q/f (L/hr)
**R101**	0.10	6299	10575	65.7	153
**R102**	0.10	1297	10575	75.6	305
**R103**	0.10	652	10575	70.1	325
**R104**	0.10	3001	10575	85.5	427
**R105**	0.10	598	10575	75.5	702
**R106**	0.10	430	10575	60.3	267
**R107**	0.10	666	10575	40.8	129
**R108**	0.10	1104	10575	38.7	217
**Geomean**	0.10	1148	10575	61.8	276
**%CV**		114		30	59

Table S3 Individual Bayesian *post hoc* estimates for the PK observed in 8 individuals who were administered 800 mg ferroquine in the human challenge study.

Results from file patab40

A visual predictive check demonstrated that the 2 compartment model was sufficient in describing PK data to 240 hours (Figure S5).

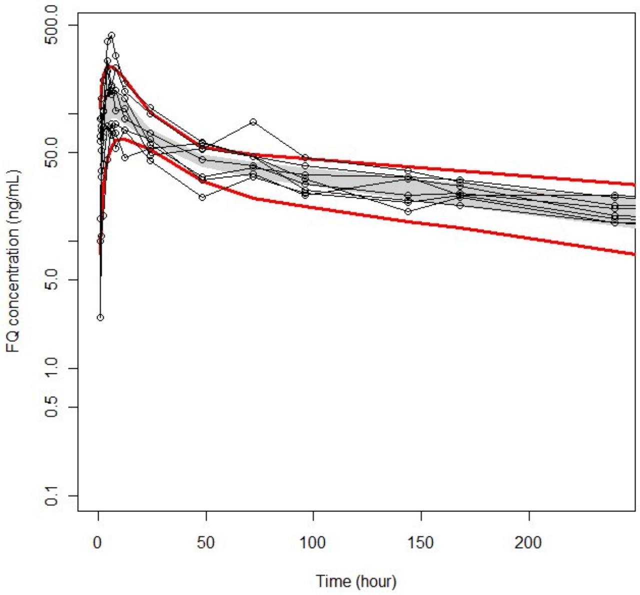

Figure S5 A visual predictive check of the model's ability to reflect the data
Observed data = points + time black lines
Predicted 0.05 and 0.95% prediction quantiles = red lines
Predicted 0.25 and 0.75% inter-quantile range = grey shading

Integrating the effects of the active metabolite

Ferroquine concentrations were used as a surrogate for metabolite concentrations within this analysis. The contribution of the metabolite on efficacy cannot be assessed. The relative efficacy of the metabolite varies between reports showing similar IC_{50} as compared to Ferroquine based on *in vitro* results or 4-fold higher IC_{50} based on clinical isolates. The ratios of parent to metabolite do change during the study although the impact should be minimal (Figure S8):

- After 800 mg, up to 100 hours the ratio is <1. However, FQ concentrations are >MPC (estimated) so maximum response is expected and FQ levels are a surrogate for the combined effective concentrations (FQ + metabolite). This assumption becomes weaker when the FQ dose is <400 mg and concentrations are not >MPC for the majority of the period and the forecasted effect may be overestimated.
- From 100 to ~400 hours the ratio does not significantly deviate from 1. The FQ concentrations can act as a surrogate for the combined effective concentrations.
- From ~400 hours onwards the ratio increases up to 1.6 on average at 678 hours. Use of FQ levels will underestimate the combined effective concentrations and may underestimate the efficacy during this period. This magnitude of the effect is difficult to estimate because relative efficacy of the metabolite ranges from 0.3 to 1 equivalence with FQ and would only be apparent when [FQ]<MPC but [Met]>MPC and parasites remain.

This assumption would underestimate the contribution of the metabolite at doses when ferroquine levels have dropped significantly below the LOQ or MPC when the metabolite would linger (when the ratio increases). However, this would also require high doses of ferroquine so that the metabolite levels are effective and above the MPC.

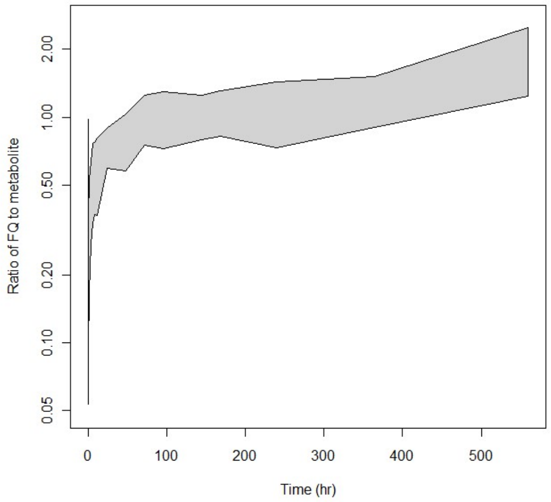

Figure S6 The ratio of metabolite levels divided by ferroquine concentrations for all subjects administered 800 mg ferroquine in the human challenge model.

N = 8, grey shading reflects the 5, 95% CI.

Modelling ferroquine human challenge model pharmacodynamics

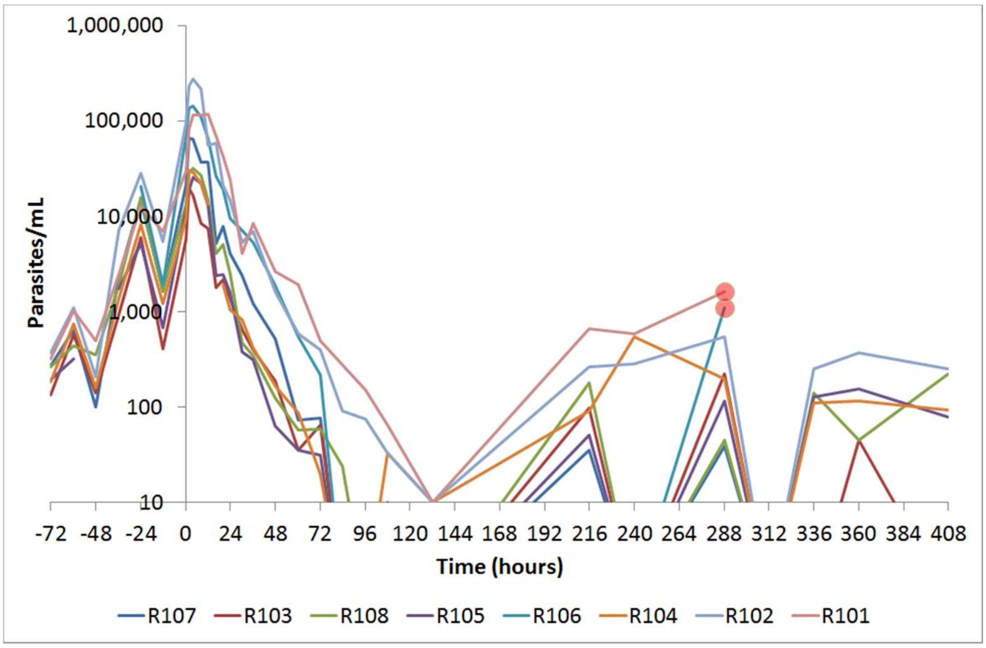

Figure S7 Volunteer parasitemia observed in the human challenge model prior to and following dosing with 800 mg ferroquine. Volunteers were inoculated at -168 hours. Ferroquine was administered at t=0.

BLOQ = 10 parasites/mL. N = 8, Riamet treatment marked with the transparent red marker

Administration of 800 mg ferroquine led to a reduction of parasitemia in the human challenge model (Figure S7).

The aim of this section was to compile a PK/PD model describing the link between ferroquine PK and its effect on parasitemia reduction (PD).

1.1 Initial parameter estimation

Initial parameter estimates were determined prior to fitting a PK/PD model. Three sequential steps were undertaken:

1. Estimation of the parasitemia growth and death rates
2. Estimation of the MPC, and
3. Validation of initial estimates through trial simulation.

1.1.1 A pharmacodynamic model for estimation of growth and death rates

A pharmacodynamic model was compiled to estimate growth and clearance of parasitemia. The data were categorized into pre- and post- dosing effect where the time point of dosing effect was determined in the modelling process. The PD data were truncated to the point where parasitemia was BLOQ or to include evidence of a significant trend away from the log-linear reduction in parasitemia (where the log-linear decline is no longer log-linear and is instead horizontal).

The modelling was performed in Nonmem using the following code:

```
IF (TIME.LE.LG) THEN

DADT(1)=PG*A(1)

ELSE

DADT(1)=-PRR*A(1)

ENDIF
```

Where:

- LG = lag in drug effect
- PG = parasite growth rate
- PRR = parasite elimination rate or clearance rate
- A(1) = parasite concentration (parasite/mL)

The code states that parasitemia will expand (by PG×A(1)) prior to a lag time after which it will decline (by PRR×A(1)). Then:

```
IF (TIME.LE.LG) THEN

OB=10**(LOG10(A(1))+(COS(2*PI*((TIME-PS)/PD)))*AMP)
```

```
ELSE
OB=A(1)+GM
ENDIF

Y        = IPRED*(1+EPS(1))
```

Where:

- PS = cosine phase
- PD = cosine period
- AMP = amplitude
- GM = fixed gametocyte levels
- Y = observed parasitemia
- IPRED = mixed effect parasitemia observation
- EPS = measurement error

The code states that as parasitemia grows it will display a cosine function and that as it declines a fixed level of gametocyte-derived parasitemia signal will be apparent.

This addition of the cosine function caused a significant reduction in objective function and gave a closer approximation to the observed data. The data did not support a cosine function in the parasite clearance period (post-dose effect), illustrating that there was little or no evidence of cyclical kinetics after administration of FQ.

The addition of a floor of parasitemia (called gametocyte level in recognition that the majority of parasites detected were gametocytes determined by PCR and lack of response to A/L) led to a significant reduction in objective function, improving the fit to the data (Figure S8). Average and individual parameters were estimated describing the growth kinetics and maximum decline in parasitemia (Table S4 and Table S5).

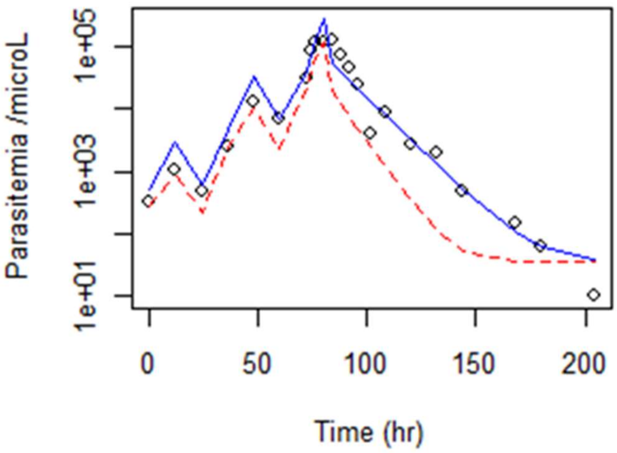

Figure S8 Observed parasitemia from subject R101 overlaid with individual and population predictions.
Time starts at first post-inoculum detection of parasitemia with dosing occurring at 72 hours.
Observed data = points
Individual prediction of parasitemia time course = solid blue line
Population prediction of parasitemia time course = dashed red line

Parameter	Units	Value (SE)	95% CI
**Obj**		2419	
**Growth rate**	1/hr	0.064 (0.004)	(0.058 - 0.071)
**Baseline parasitemia**	Parasites/mL	170 (32)	(117 - 224)
**Clearance rate**	1/hr	0.11 (0.009)	(0.10 - 0.13)
**Lag in drug effect**	hr	81 (0)	(81 - 81)
**Period**	hr	37 (0.6)	(35.7 - 37.6)
**Phase**	hr	7.0 (0.6)	(5.9 - 8.1)
**Amplitude**		0.59 (0.04)	(0.53 - 0.66)
**Gametocyte level**	Parasites/mL	36 (7)	(24 - 47)

Table S4 Parameter estimates for the fitting of the PD model to the ferroquine human challenge model parasitemia data. N=8, model = run102.mod, method=first order conditional estimation with interaction in Nonmem VII, confidence intervals estimated from bootstrap conducted with 3000 replicates using PsN and Nonmem.

ID	Growth rate (1/hr)	Baseline (Parasite/mL)	Reduction (1/hr)	Lag time (hr)	Period (hr)	Phase (hr)	Amplitude
**R101**	0.064	432	0.08	81	37	8.4	0.59
**R102**	0.064	428	0.09	81	37	5.5	0.59
**R103**	0.064	67.9	0.12	81	37	7.6	0.59
**R104**	0.064	95.2	0.13	81	37	7.4	0.59
**R105**	0.064	75.7	0.15	81	37	6.4	0.59
**R106**	0.064	330	0.09	81	37	5.8	0.59
**R107**	0.064	168	0.11	81	37	6.8	0.59
**R108**	0.064	143	0.15	81	37	8.1	0.59
**Geomean**		**171**	**0.11**			**6.9**	
**%CV**		**81**	**23**			**15**	

Table S5 Individual Bayesian *post hoc* estimates for the PD observed in 8 individuals who were administered 800 mg ferroquine in the malaria human challenge study. From file patab102

The clearance rate of 0.11 (0.10 - 0.13) /hr is equivalent to a $\log_{10}$ reduction ratio of 2.3 (2.1 - 2.7) per 48 hours. The lag in drug effect is from time of first detected parasites (72 hours before dosing) so the lag in observed drug effect is 9 hours (81 - 72 hours).

The population and individual estimates of the parasitemia growth and clearance characterize the intrinsic parasite kinetics and the maximal drug effect. Estimation of an MPC / MIC would link the above parameters to ferroquine concentrations.

1.1.2 Estimation of MPC

The MPC is the concentration at which parasite killing decreases from a maximal process (characterized by a log-linear decrease in parasitemia). The MPC defines these observations:

- **Recrudescence:** If drug concentrations drop below the MPC (and MIC) before parasitemia is cleared then the parasite population will grow (at the growth rate) and recrudescence will occur.
- **Treatment:** If drug concentrations are maintained above the MPC then parasitemia will reduce in a log-linear fashion until parasitemia is cleared (or recrudescence occurs as described above)

Observations of recrudescence mean that concentrations equivalent to the MPC will have been observed before or around the nadir of parasitemia. Observations of treatment mean that drug concentrations were above the MPC until or after the theoretical total clearance of parasites.

Administration of ferroquine in the human challenge study led to two subjects recrudescing and the remaining six subjects being successfully treated. These observations provided data with which to estimate the MPC of ferroquine in the human challenge model.

Recrudescent subject MPCs

Subjects R101 and R106 recrudesced and were treated with A/L at 288 hours following dosing with ferroquine. The parasitemia of both subjects responded to A/L treatment, providing evidence that the recrudescence was an upsurge in asexual parasites rather than of gametocytes. Gametocytemia was characterized with levels equivalent to approximately 30 parasites/mL or less.

Subject R101 had an observed nadir of parasitemia at 132 hours after ferroquine dosing (dosing occurred 72 hours after first post-inoculum detection of parasites), coinciding with ferroquine concentrations of 31 ng/mL at 96 hours and 17 ng/mL at 144 hours. The 132 hour concentration was estimated using a PK profile modeled from individual PK parameter estimates for subject R101 and was 22.7 ng/mL.

The time of parasitemia nadir for subject R106 had to be inferred from the subject's parasitemia kinetics (Figure S9). The parasitemia growth and parasite rates were determined by PD modelling (Table S5). The growth rate was 0.0643 /hr (an expansion of $10^{1.34}$-fold every 48 hours) and the PRR was 0.0935/hr (a contraction of $10^{1.95}$-fold every 48 hours). Growth curves were determined that would reach parasitemias of 1099 and 10 parasites/mL at 288 and 240 hours respectively. The former reflects the sole parasitemia observation before Riamet intervention. The latter reflects the LOQ that the parasitemia must have been equal or less than at the time point preceding the sole observation.

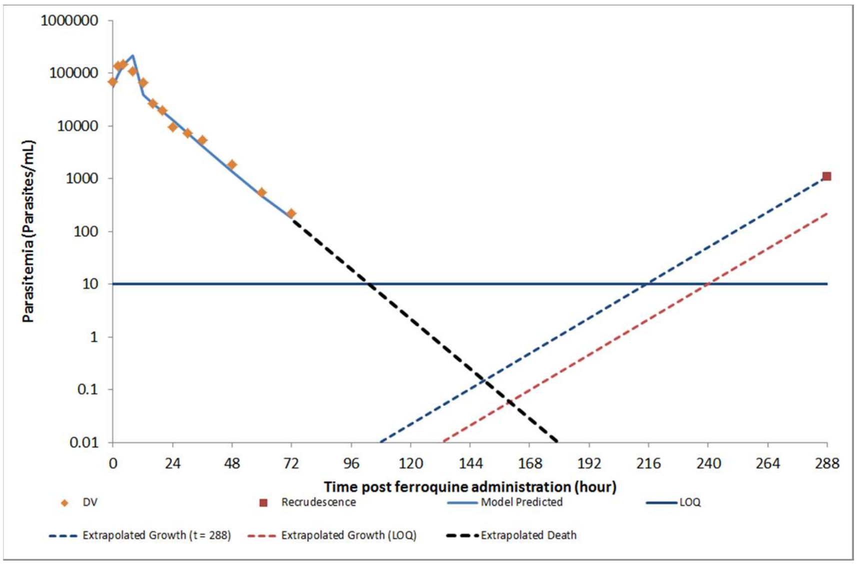

Figure S9 An illustration of the estimation of subject R106's nadir of parasitemia

- Observed data = points (t=0 hr - t=72 hr: decline in parasitemia, t=288 hr: recrudescence)
- Model-predicted decline in asexual parasitemia = blue line
- Extrapolated decline in parasitemia = black dashed line
- Predicted growth (recrudescence) curves = blue dashed (based on recrudescence value), red dashed (based on LOQ at t=240 hr)
- LOQ = blue horizontal line (10 parasites/mL)

The intercepts of the two growth curves with the death curve inferred that the estimated parasitemia nadir occurred between 149.5 and 160.0 hours at 0.15 to 0.06 parasites/mL respectively. The coincidental ferroquine concentrations at these time points are approximately 26.4 and 25.2 ng/mL respectively.

MPC inference from non-recrudescent subjects

The non-recrudescent asexual parasitemia curves can be extrapolated to determine when parasitemia crosses a threshold for recrudescence (0.003 parasites/mL equivalent to 15 parasites/total blood in a 70 kg subject – estimated from earlier challenge studies) or reaches 1 parasite/total blood in a 70 kg subject. In all cases the linear decline determined by model-estimated PRR was assumed, and used to extrapolate the asexual parasitemia decline. An estimated MPC would be equal to or less than the coincidental concentration of ferroquine observed at the same time.

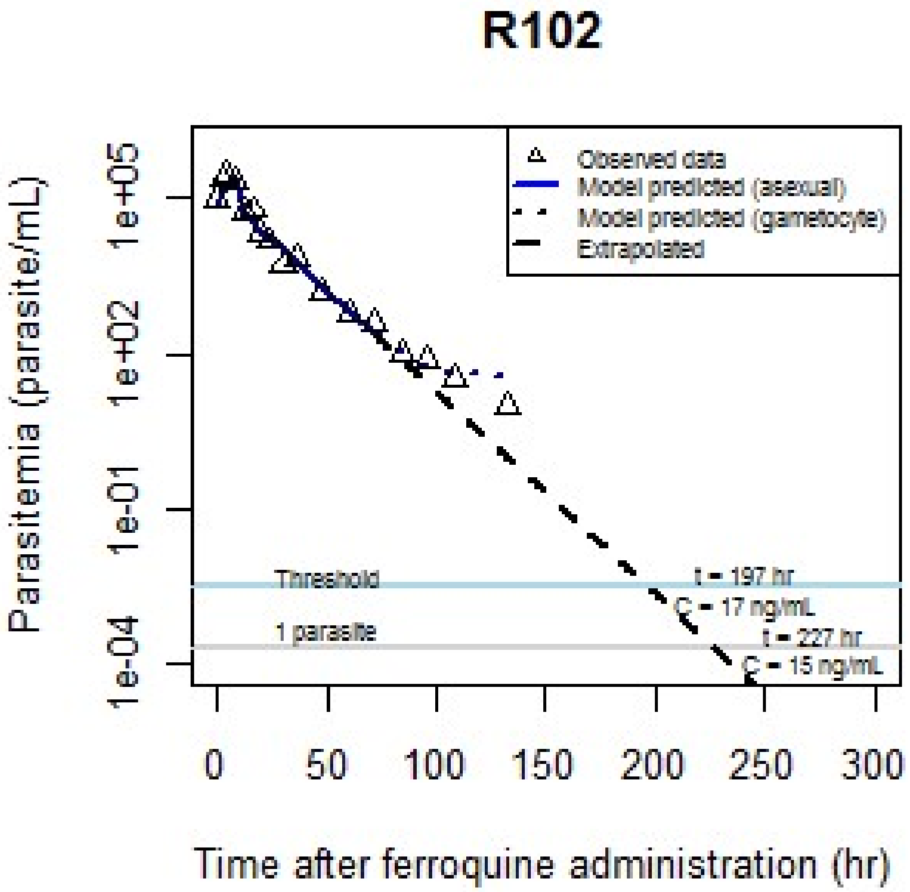

Figure S10 Using extrapolation of the drug effect on asexual parasitemia to estimate time to complete and effective parasite clearance.
LOQ = 10 parasites/mL

In the example shown (Subject R102, Figure S12), the model predicted asexual parasitemia (using the model-estimated PRR) is used to extrapolate beyond the start of the period when parasitemia is comprised of asexual and gametocyte parasites. The time when the extrapolated parasitemia intersects the recrudescence threshold and when it is equivalent to 1 parasite/total blood is noted. In this example the times are 197 and 227 hours respectively. The ferroquine concentrations at these times are 17 and 15 ng/mL.

The approximate MPC estimated for subject R102 is less than or equal to a ferroquine concentration between 15 and 17 ng/mL.

Subject	Time (hr)		Concentration (ng/mL)	
	Threshold	1 Parasite	Threshold	1 Parasite
**R102**	197	227	17.3	14.8
**R103**	133	155	26.3	23.4
**R104**	129	150	23.9	21.2
**R105**	115	134	31.2	27.8
**R107**	153	177	28.7	26.9
**R108**	115	133	28.8	27.7
**Median**	131	153	27.5	25.2
**5,95% CI**	115-186	133-215	19-31	16-28

Table S7 The time taken for parasitemia decrease to an extrapolated intercept with either a threshold of 0.003 parasites/mL or 0.0002 parasites/mL (equivalent to 1 parasite per 70kg subject) and the equivalent concentration of FQ concurrent with these times.

The extrapolation (Table S6) indicated that the T>MPC on average had to be greater than 142 hours (5, 95% CI = 115-211 hours). This translated into an average concentration that the MPC would be less than: 26.6 ng/mL with a range (5, 95% CI) spanning 16 - 30 ng/mL.

MPC estimation

A value and range for MPC / MIC was chosen for simulation as 20 ng/mL (with an arbitrary %CV=0.2) reflecting inter-individual variance and uncertainty in the estimation process. The value was chosen because it met these criteria:

- That the two observed MPC values (22.7 and 25-26 ng/mL) are above the predicted median MPC (20 ng/mL), reflecting that only 2/8 recrudesced. If the predicted MPC median was higher (equal to or higher than the two observed MPC values) then more recrudescences should have been observed.
- That the range go below 15 ng/mL, reflecting the lower range of concentrations that the MPC must be below.

Additional median MPC values ranging from 18-22 ng/mL were tested and validated against trial simulations of the human challenge model.

Human challenge model population estimate of MPC
**20 ng/mL (%CV = 20)**

1.1.3 Trial simulations and validation of MPC

The population estimates of parasitemia growth and death rates and MPC were used to simulate the human challenge model. Successful simulation would provide confidence that the parameters reflected ferroquine's effect on parasitemia.

Success in the trial simulations was determined by which point was achieved first:

- Ferroquine concentrations decreasing below MPC: outcome = recrudescence
- Forecasted parasitemia declines to 1 parasite/body: outcome = successful treatment

Human challenge model trial simulations

The human challenge model was simulated using the PD model:

$$\frac{dP}{dt} = P \times \left(G - D \times \frac{C(t)^H}{C(t)^H + IC_{50}^H} \right)$$

$$D = D_{max} \times (1 - \exp^{-k \times t})$$

Where: P = parasites (parasite/mL)

G = 1st order growth rate

D = 1st order parasite reduction (or death) rate

C = drug concentration

IC_{50} = drug concentration causing parasite reduction that is 50% of maximum

H = slope coefficient of response slope

D_{max} = maximum parasite reduction rate

k = exponential factor determining rate of change of parasite reduction rate

Synchronous kinetics will be incorporated:

$$log_{10}(P_t) = log_{10}(P') + \left[\cos\left(2\pi\times\frac{\text{Time-Phase}}{\text{Period}}\right)\right]\times\text{Amp}$$

Where: P' = Underlying asynchronous parasitemia

The PD model was applied using the population estimates of the 2 compartment PK model parameters and MPC estimates transformed to IC50 (Figure S11). The time to effective parasite clearance was determined for each simulated subject up to Day 14 following FQ single dosing. The subject was said to recrudesce if the time to effective parasite clearance exceeded the time above MPC determined from the simulated PK - this was tested out to day 14 with observations of recrudescence able to occur beyond day 14. The trial with n=8 subjects was repeatedly simulated 1000 times and the average successful treatment rate determined. The treatment rate observed was 0.75 on Day 14 in the human challenge study and the median predicted rate was 0.75 on Day 14 (5, 95%CI = 0.49-1.0). The treatment rate could be considered to be constant from day 10 onwards as any recrudescence would have already occurred and there was no opportunity for reinfection.

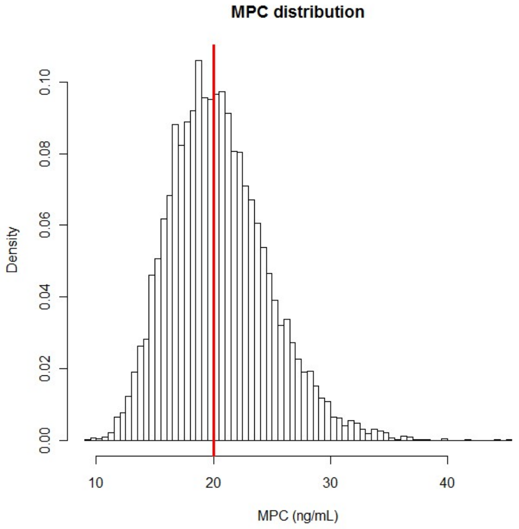

Figure S11 A density histogram showing the distribution of MPC values when median MPC = 20 ng/mL with log-normal distribution and a %CV=0.2.

An MPC of 20 ng/mL (%CV=0.2) was validated from the trial simulations where MPC ranged from 5- 50 ng/mL. The trial simulations demonstrated that, in combination with the 2 compartment PK, the MPC=20 ng/mL provided a treatment rate of 0.75 the same as observed in the trial (Figure S10 and Figure S13).

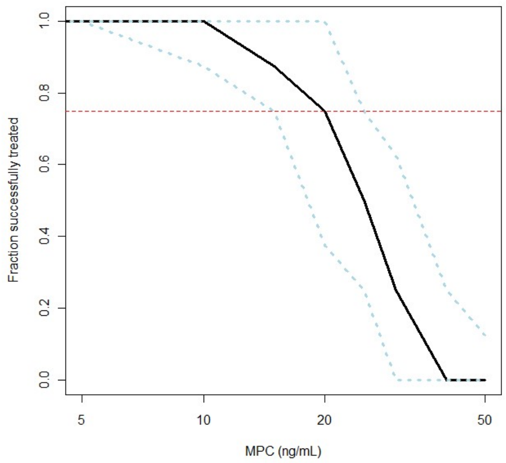

Figure S12 The effect of MPC on the predicted fraction of subjects in the human challenge study who were successfully treated as defined in the above text.

N=8/trial, 1000 replicates

Prediction intervals = dashed blue line

Observed treatment rate = horizontal dashed red line

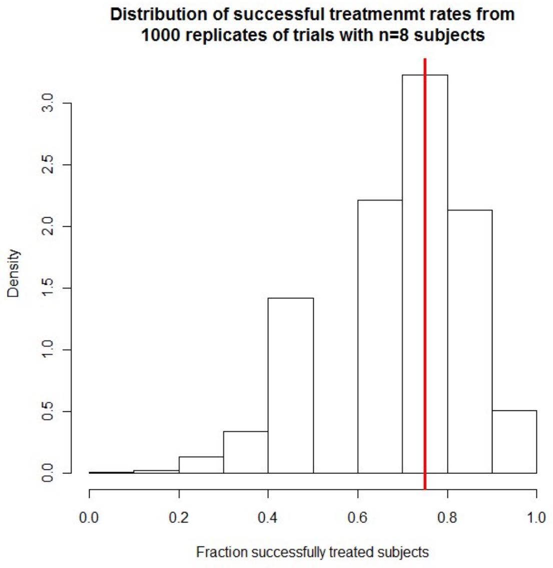

Figure S13 The distribution of successful treatment fractions from simulated human challenge study simulations with median MPC=20 ng/mL with log normal distribution and a %CV=0.2

N=8/trial, replicates = 1000

Observed treatment rate = vertical red line

The trial simulation demonstrated that 20 ng/mL was an adequate estimate of MPC for the purposes of replicating the data using the PK/PD model with the individual volunteer's data. Fitting of all the data with the PK/PD model failed possibly reflecting the paucity of recrudescence data which had necessitated the estimation of MPC values or ranges by first principles.

2 Appendices

2.1 Challenge model ferroquine PK

Time (h)	R101	R102	R103	R104	R105	R106	R107	R108
0.5	2.5	15	67	10	61	91	2.5	2.5
1	11	52	93	32	75	133	80	36
2	16	105	103	40	79	185	105	66
4	44	147	144	80	80	218	369	259
6	84	168	149	73	68	142	416	162
8	70	105	153	54	84	154	289	231
12	45	109	92	75	74	131	175	149
24	54	47	71	43	63	58	111	100
48	59	54	44	22	30	32	60	53
72	46	46	39	32	34	38	46	87
96	31	25	28	24	23	33	39	45
144	17	21	23	20	31	32	32	36
168	22	19	24	23	27	23	30	29
240	14	14	18	15	16	23	22	19
364	11	13	18	13	13	18	18	21
560	9	10	9	7		12	16	15

2.2 Challenge model SSR97213 PK

Time (h)	R101	R102	R103	R104	R105	R106	R107	R108
0.5		3	8	3	3	6	3	
1		6	36	8	22	35	11	3
2	3	18	63	12	30	52	34	11
4	10	59	97	29	43	81	196	144
6	28	84	118	25	35	63	300	74
8	28	62	120	19	55	79	220	163
12	16	90	60	29	51	75	137	106
24	36	37	65	25	52	36	94	81
48	60	48	44	11	25	23	63	49
72	40	48	43	24	26	29	61	77
96	22	30	37	18	22	28	49	47
144	15	25	29	15	31	29	39	37
168	20	24	31	18	28	26	38	38
240	14	14	18	15	18	26	22	19
364	11	13	18	13	15	28	18	21
560	9	10	9	7		23	16	15

2.3 Challenge model parasitemia

Time (h)	R101	R102	R103	R104	R105	R106	R107	R108
-72	321	367	135	185	194	979	269	263
-60	1,033	1,109	570	750	323		647	437
-48	493	207	140	157		129	100	357
-36	2,476	7,082	935	1,355	1,749		2,353	1,892
-24	12,821	28,062	6,054	8,292	5,173	20,834	14,828	15,900
-12	6,874	5,484	413	1,229	682	1,913	1,653	1,619
0	29,976	94,543	5,739	9,608	11,423	68,611	21,306	13,209
2	82,767	231,177	19,870	30,531	18,582	138,155	66,440	28,426
4	115,573	274,999	16,574	28,888	25,649	144,733	64,536	31,562
8	114,029	215,457	8,353	22,048	22,430	109,454	37,024	27,039
12	119,182	55,881	7,544	13,274	13,182	65,948	36,777	14,829
16	68,543	58,558	1,784		2,365	26,024	5,214	4,065
20	43,032	21,084	2,160	1,926	2,438	19,144	7,825	5,010
24	24,632	14,815	1,364	1,039	1,585	9,546	4,122	2,519
30	4,089	5,364	643	817	379	7,122	2,378	475
36	8,372	6,952	395	421	315	5,280	1,208	346
48	2,664	1,611	188	165	64	1,868	515	124
60	1,907	583	36	87	35	548	74	58
72	495	399	65	20	32	217	77	59
84	BLOQ	92	BLOQ	BLOQ	BLOQ	BLOQ	BLOQ	24
96	150	75	BLOQ	BLOQ	BLOQ	BLOQ	BLOQ	BLOQ
108	64	33	BLOQ	33	BLOQ	BLOQ	10	BLOQ
132	10	10	BLOQ	10	BLOQ	BLOQ	BLOQ	BLOQ
216	664	262	98	90	51	BLOQ	35	179
240	586	281	BLOQ	552	BLOQ	BLOQ	BLOQ	BLOQ
288	**1,605**	549	223	197	115	**1,099**	39	46
312		BLOQ	BLOQ	BLOQ	BLOQ		BLOQ	BLOQ
336		250	BLOQ	110	129		BLOQ	139
360		372	45	116	155		BLOQ	45
408		250	BLOQ	93	79		BLOQ	221

Parasitemia values highlighted in bold designate parasitemia just prior to Riamet intervention

2.4 Challenge model pfs25 gametocyte PCR transcripts

Time (h)	R101	R103	R104	R107	R108
96	6				
120				397	
144	423	213			335
168	939	4711	1880	2198	1767
216	57596	9254			7337
288	**279749**	26215			24711
360	8327	17903			32355
408		3068			8547
456		7520			1086

Transcript values highlighted in bold designate parasitemia just prior to Riamet intervention

Appendices B - modelling plots and code

1 Individual PK plots; "1", "2" etc. corresponds to subjects R101, R102, etc.

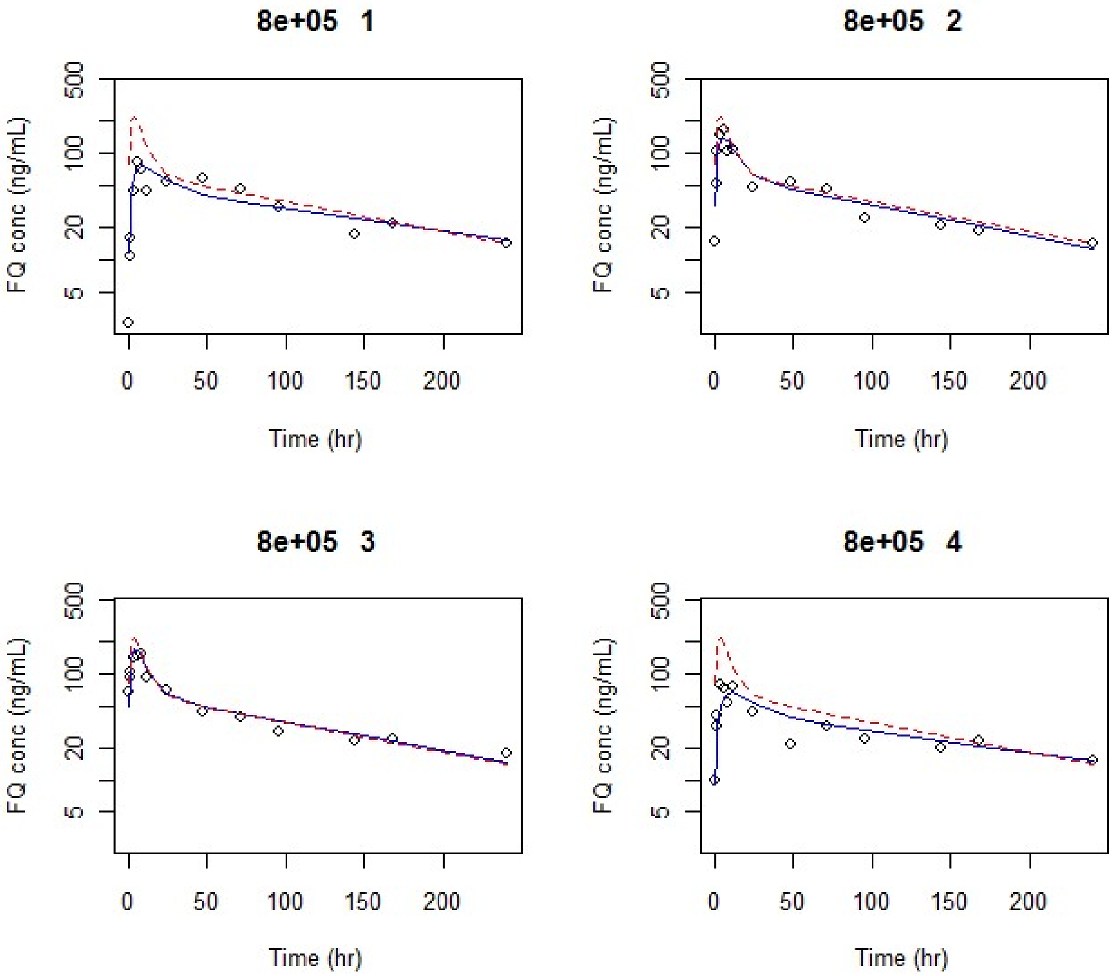

Figure S14 Observed PK of subjects 1-4 overlaid with individual (blue line) and population (red dashed line) predictions.

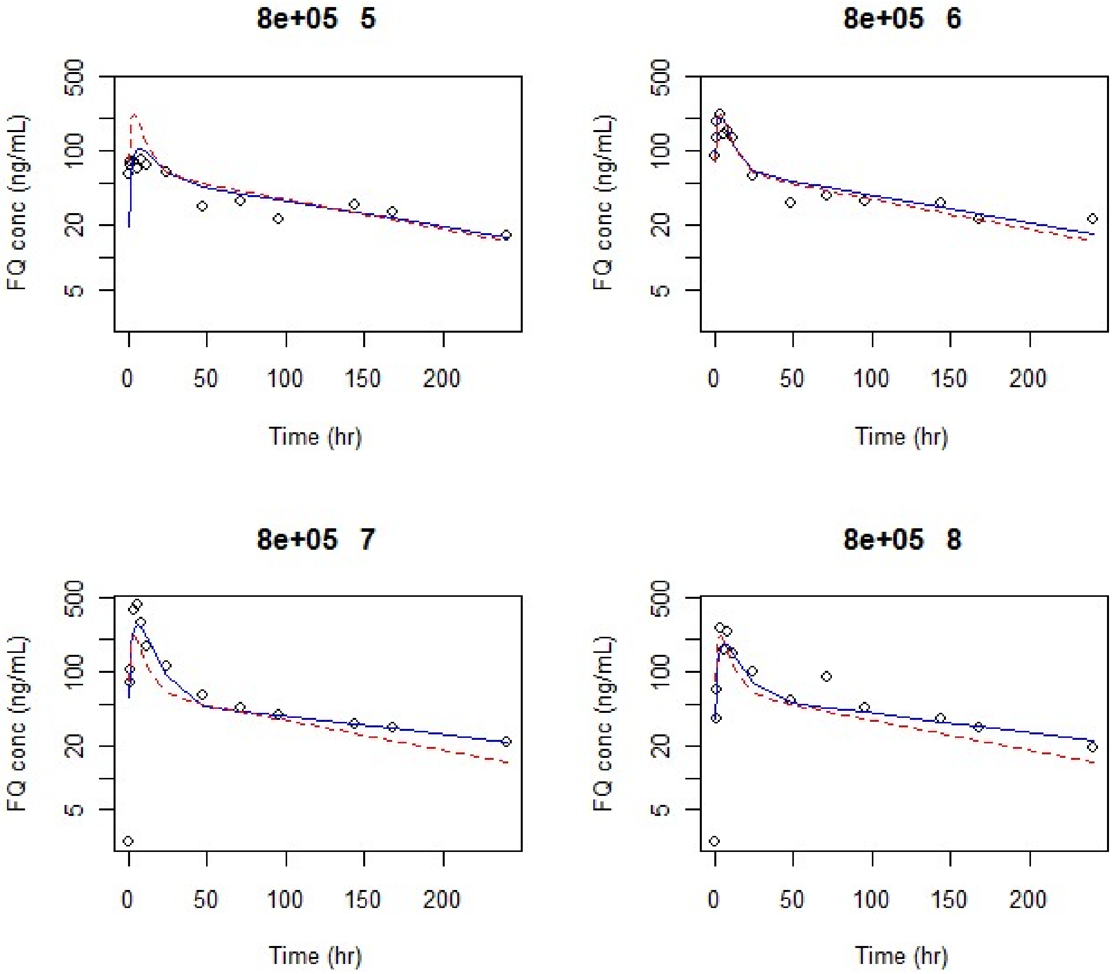

Figure S15 Observed PK of subjects 5-8 overlaid with individual (blue line) and population (red dashed line) predictions.

2 Individual PD plots; ; "1", "2" etc. corresponds to subjects R101, R102, etc.

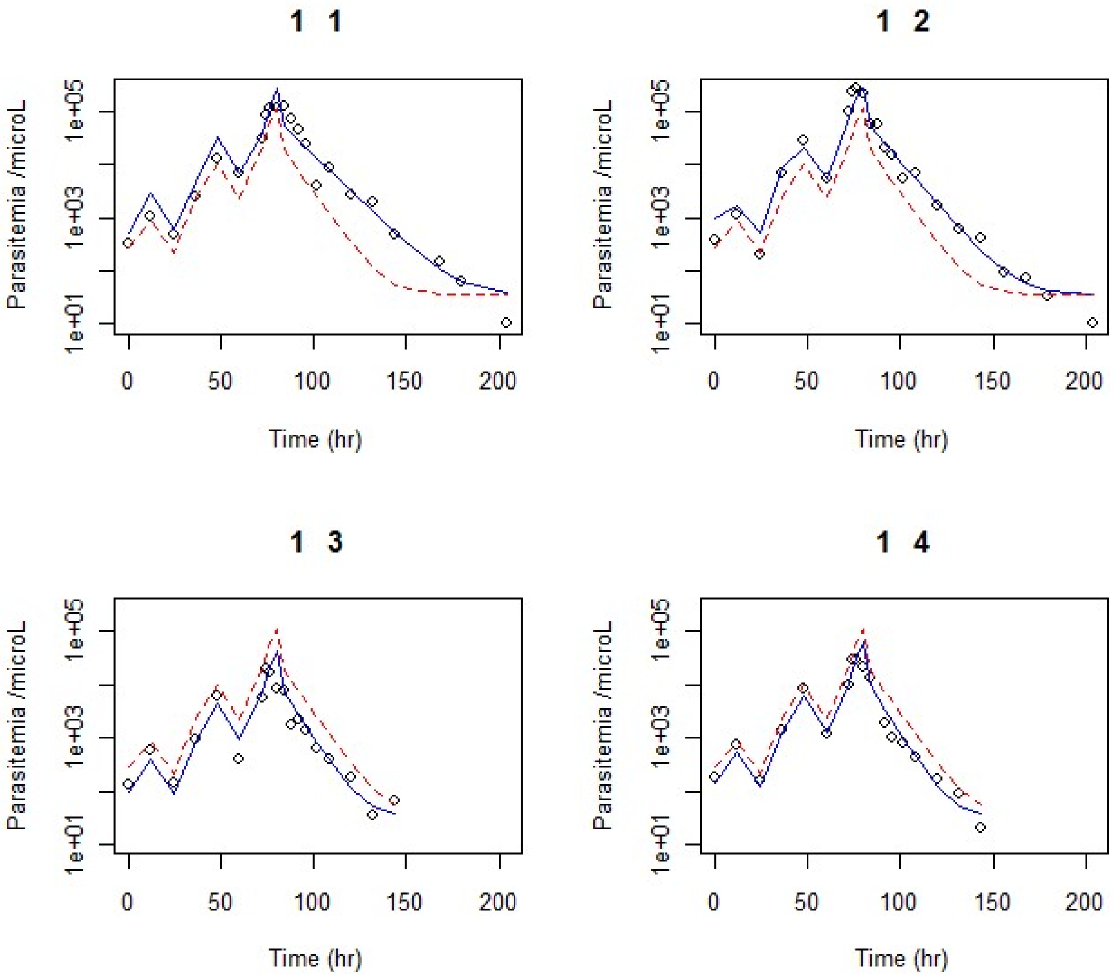

Figure S16 Observed PD of subjects 1-4 overlaid with individual (blue line) and population (red dashed line) predictions.

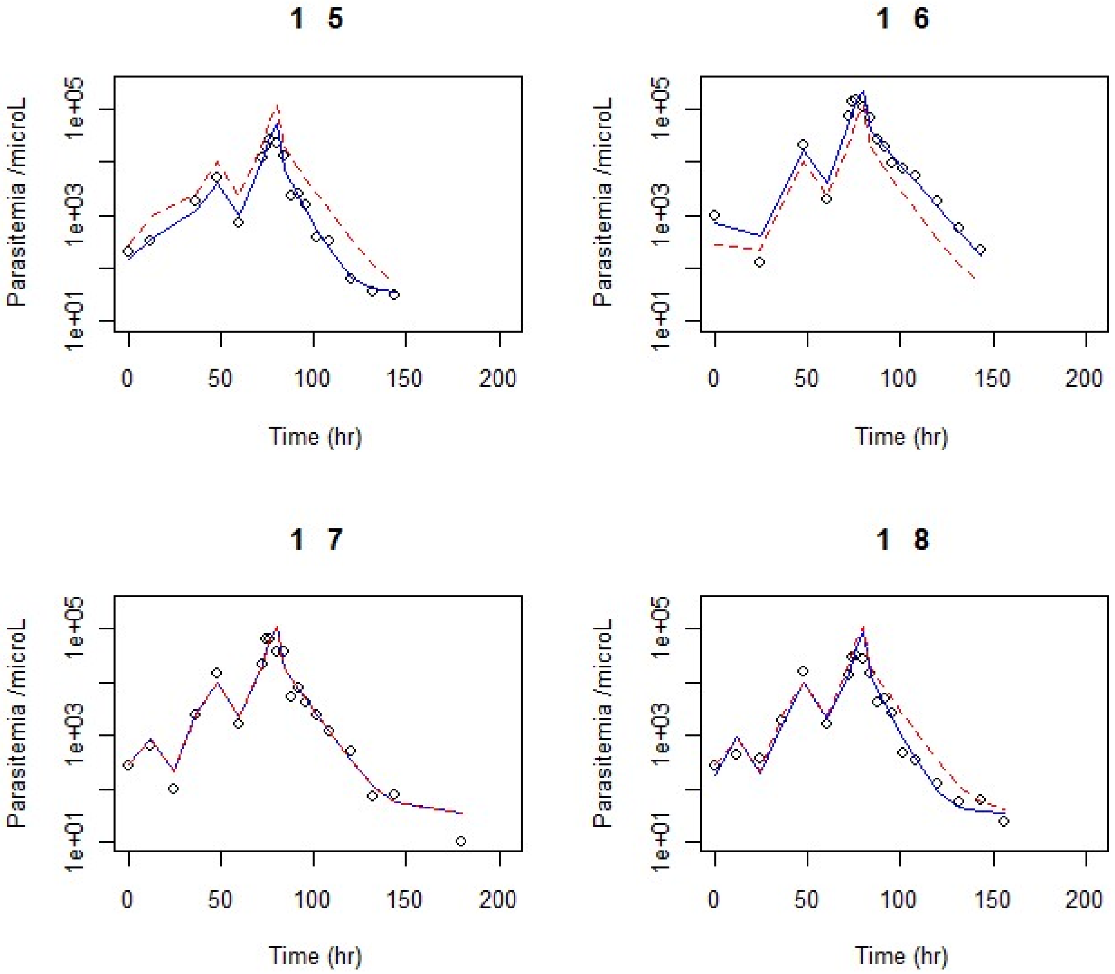

Figure S17 Observed PD of subjects 5-8 overlaid with individual (blue line) and population (red dashed line) predictions.

3 Individual PD regression plots; ; "1", "2" etc. corresponds to subjects R101, R102, etc.

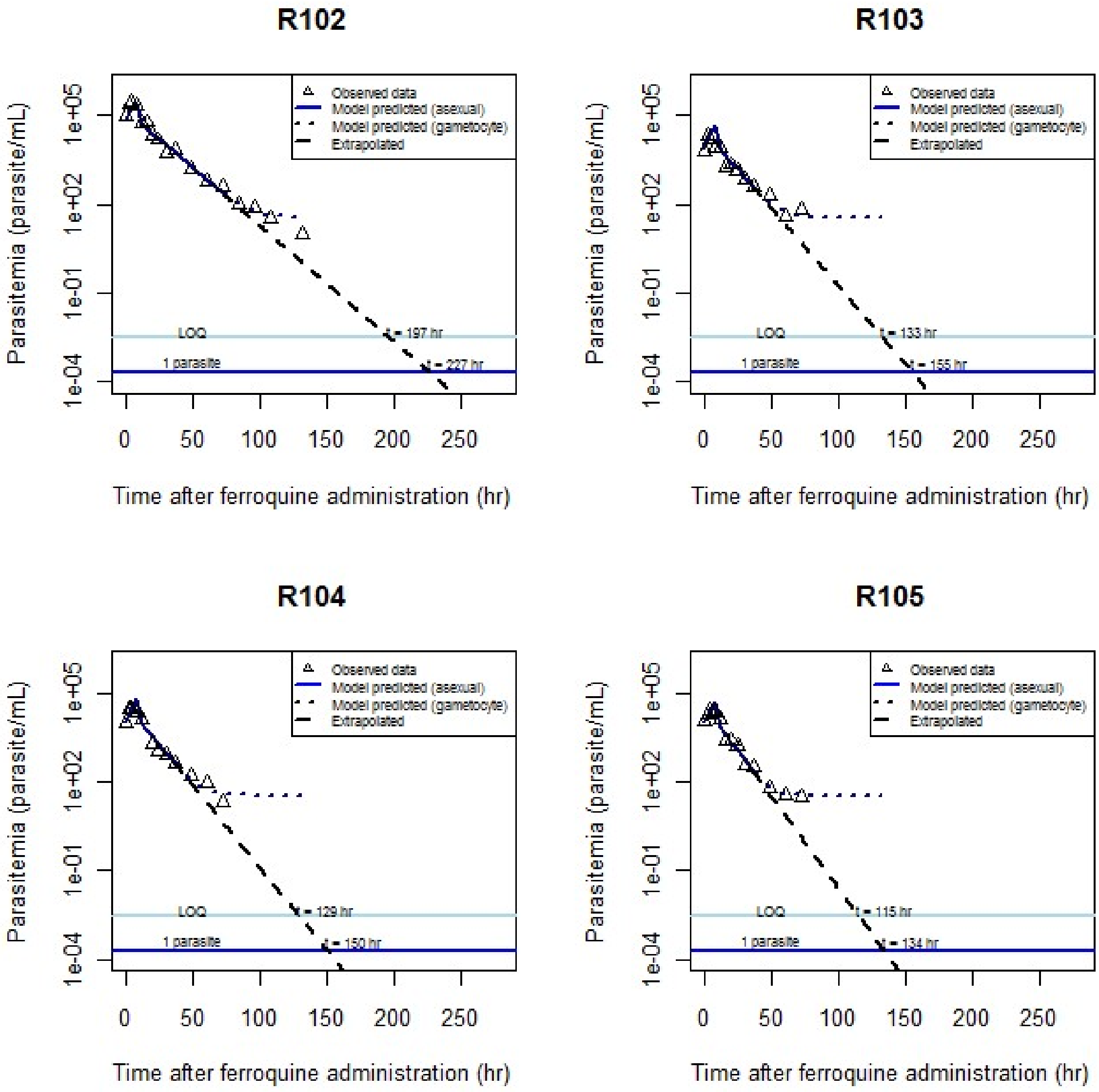

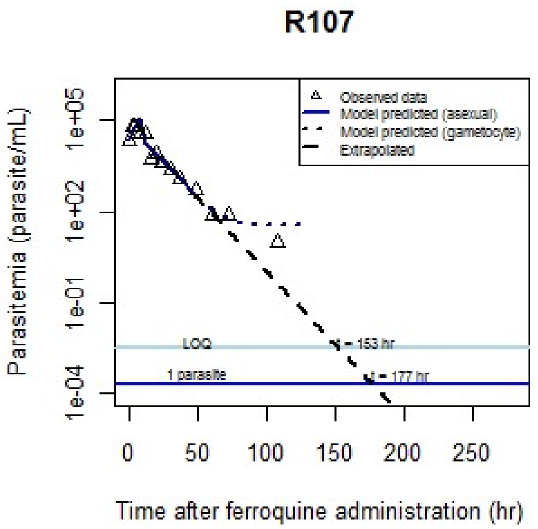

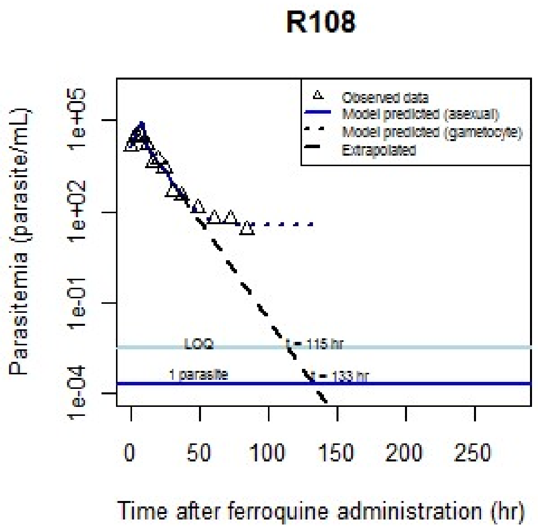

Reference

1. Savic RM, Jonker DM, Kerbusch T, Karlsson MO: **Implementation of a transit compartment model for describing drug absorption in pharmacokinetic studies.** *J Pharmacokinet Pharmacodyn* 2007, **34:**711-726.
